# Design assessment and preliminary performance evaluation of a novel device for non-invasive emergency treatment of acute respiratory failure

**DOI:** 10.1007/s13534-026-00619-3

**Published:** 2026-10-25

**Authors:** Federica Ciceri Negri, Eleonora Salurso, Giuseppe Giardina, Filippo Consolo, Giovanni Landoni, Alberto Cesare Luigi Redaelli, Gianfranco Beniamino Fiore

**Affiliations:** 1 Department of Electronics, Information and Bioengineering, Politecnico Di Milano, Milan, Italy; 2 Department of Anaesthesia and Intensive Care, IRCCS San Raffaele Scientific Institute, Milan, Italy; 3 Faculty of Medicine and Surgery, Vita-Salute San Raffaele University, Milan, Italy

**Keywords:** Acute respiratory failure (ARF), Non-invasive ventilation (NIV), Continuous positive airway pressure (CPAP), Positive end-expiratory pressure (PEEP), Pre-hospital emergency care

## Abstract

Acute respiratory failure (ARF) requires rapid intervention to prevent patient deterioration and life-threatening complications. Early respiratory support through prehospital Continuous Positive Airway Pressure (CPAP) has been shown to improve ARF outcomes. However, existing CPAP devices present technical barriers for use in out-of-hospital emergency scenarios. This study presents the design and preliminary performance evaluation of a novel portable, ready-to-use CPAP device for out-of-hospital respiratory support. The device includes a blower providing steady air insufflation through a face mask, with Positive End-Expiratory Pressure (PEEP) regulated by two fixed-point PEEP valves. To identify optimized operational conditions ensuring target respiratory support, a lumped-parameter model (LPM) was developed to simulate device–patient interaction. Input data for the LPM included experimental characteristics of the device components. Simulations were conducted using breathing profiles representing varying ARF-like conditions and severity. Additionally, computational fluid dynamics (CFD) analysis was performed to assess the risk of CO_2_ rebreathing through the mask. LPM simulations described the dynamic interaction between the breathing profile and device performances, and the consequent pressure and flow patterns during the respiratory cycle. Across all simulated conditions and device settings, PEEP remained close to the predefined target of 5 cm H_2_O without reaching critical thresholds, confirming design choices efficacy. CFD analysis indicated an end-expiratory residual CO_2_ concentration of ~2%, suggesting a low risk of rebreathing between cycles. Our results confirmed the validity of our design solutions and suggest a potentially safe and efficient profile as a promising solution for early CPAP use in emergency settings.

**Supplementary Information:**

The online version contains supplementary material available at https://doi.org/10.1007/s13534-026-00619-3.

## Introduction

Acute respiratory failure (ARF) is a life-threatening clinical condition in which the respiratory system fails to maintain adequate gas exchange. Timely initiation of respiratory support is a key determinant of ARF patient outcomes, as the delay in treatment is correlated with increased risk of progression to invasive ventilation and mortality.

The first-line management of ARF primarily involves non-invasive respiratory support to improve oxygenation and reduce respiratory muscle fatigue. In particular, continuous positive airway pressure (CPAP) represents a well-established, safe, and effective therapy to prevent airway collapse, promote alveolar recruitment and decrease the work of breathing [1, 2]. Of note, accumulating evidence from both observational studies and systematic reviews demonstrate significant clinical benefits of early initiation, possibly in the prehospital setting, of non-invasive ventilation (NIV) via CPAP, with reported improved outcomes including reduced risk for endotracheal intubation, lower in-hospital mortality and shorter intensive care unit length of stay compared with standard care [3–7]. The clinical relevance of early application of out-of-hospital NIV was also recognized in a recent consensus paper, where a group of multidisciplinary experts recommended the initiation of treatment within 10 min of ARF presentation to both relieve symptoms and prevent clinical deterioration; importantly, the authors highlighted that NIV should be preferred over oxygen therapy in the presence of acute respiratory distress, also when diagnosis is often unclear [8].

Nevertheless, the widespread use of out-of-hospital non-invasive respiratory support remains limited by the lack of devices specifically conceived to enhance rapid deployment in uncontrolled emergency scenarios. Pre-hospital CPAP systems, such as the Boussignac CPAP, have been successfully used by emergency medical services in response to ARF [9, 10]. However, these systems are pneumatically driven and require connection to a high-flow oxygen source, with delivered pressure depending on the supplied gas flow. Existing CPAP systems designed for in-hospital use require significant expertise and medical background for setup manoeuvres and setting, and adjustment of ventilatory parameters in response to evolving patient’s conditions. Moreover, those devices depend on external power or oxygen sources. Accordingly, those devices are evidently impractical for out-of-hospital uncontrolled environments, especially for use by first responders with no medical expertise. On the other hand, even portable CPAP systems currently marketed for at-home management of sleep apnoea often entail a degree of operational complexity, that is, the setting of target pressure values or the selection of different pre-defined respiratory support profiles, thus undermining their utility in urgent out-of-hospital applications by non-experts.

In this study, we present the design characterization and a preliminary performance evaluation of a novel CPAP device. This device addresses an unmet clinical need for readiness and ease of use, enabling its prompt deployment in out-of-hospital ARF emergency scenarios.

In detail, we report our characterization process aimed at validating the rationale of component selection, establishing optimized operational conditions of the system, and conducting a preliminary evaluation of the device performance. The process included integrated experimental characterization, lumped parameter model (LPM) simulations, and numerical, computational fluid dynamics (CFD) studies.

## Materials and method

### Device overview: operating principle and components

The device is specifically designed to deliver continuous airway pressure (positive end-expiratory pressure, PEEP) to the patient’s airways, thereby providing non-invasive respiratory support. It is intended for use by first responders and potentially untrained bystanders. Accordingly, the definition of the design requirements prioritized intuitiveness and easiness-of-use while maintaining high level operational safety. A comprehensive description of the device is provided in [11].

The device’s operating principle is based on the constant action of an air blower that draws ambient air and insufflates it through an internal flow path and a standard face mask towards the patient’s airways, thereby maintaining a positive pressure at the airway openings. Limitation of such pressure is entrusted to two, differently rated, PEEP valves, properly situated along the airflow path. By design, the desired PEEP generated by the device should be at least 5 cmH_2_O to provide low-level respiratory support potentially ensure a therapeutic benefit, whereas the maximum pressure applied during the respiratory cycle should not exceed 10 cmH_2_O, to avoid excessive airway pressurization and lung overdistention, particularly in the intended unmonitored out-of-hospital setting.

**Fig. 1:**
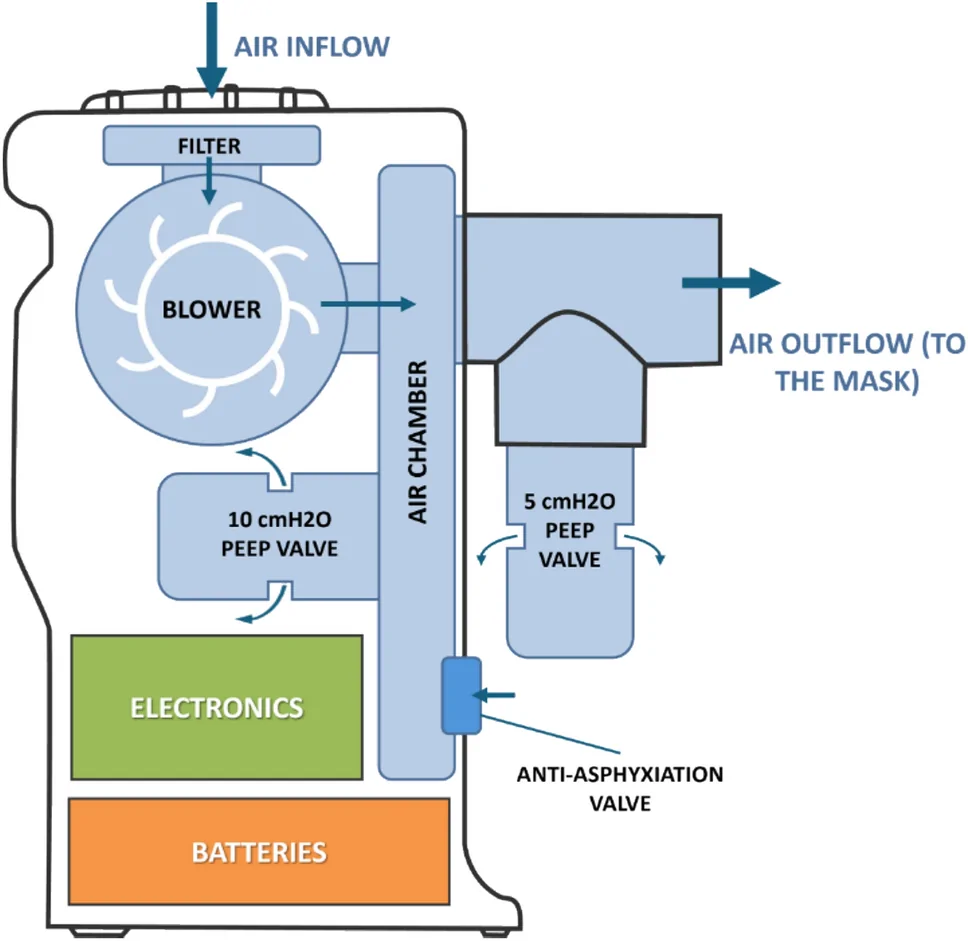
Schematic representation of the device functional components. Arrows depict the internal airflow trajectory: ambient air enters the system and passes through an anti-particulate filter placed upstream of a centrifugal blower and is then conveyed into an air-distribution chamber and eventually to the patient’s airways. Airflow pressure level is regulated via two mechanical PEEP valves and is delivered to the patient via oronasal mask

Figure 1 shows an overview of the functional components of the device, with a focus on the airflow path. Before entering the system, ambient air passes through an anti-particulate filter positioned upstream of the centrifugal blower working at a constant, design-fixed rotational speed, after which it is conveyed into an air-distribution chamber. From the air-distribution chamber, airflow is delivered to the patient via a oronasal mask. Two mechanical PEEP valves—namely an ‘external’ PEEP valve set at 5 cmH_2_O and an ‘internal’ PEEP valve set at 10 cmH_2_O—are connected to the chamber to regulate the pressure supplied to the patient’s airways. Additionally, an anti-asphyxia valve is incorporated into the chamber to ensure safe, unobstructed patient inspiration even in the event of airflow-generation system failure.

Blower selection was guided by typical performance requirements for ventilation systems, together with application-specific constraints aimed at minimizing overall dimensions, limiting current consumption and reducing manufacturing costs. The selected unit is a 12-V DC brushless blower, actuated via an external driver using PWM control. The airflow-distribution chamber was engineered to decelerate and expand the high-velocity jet generated downstream of the blower. This attenuate chaotic and turbulent flow structures and promoting more uniform flow rate and pressure patterns at the patient interface.

### Experimental characterization

We performed separate characterization to isolate the active flow-dependent behaviour of the sub-assembly deputed to deliver the main air flow (i.e., the filter–blower–air-chamber (FiBAC) sub-assembly) from the passive pressure-flow behaviour of the two PEEP valves, whose role is discharging excess airflow to keep the positive pressure within the desired range.

To evaluate the performance of the FiBAC assembly, aeraulic characterizations were conducted in a configuration where the inner and outer PEEP valves were removed and replaced with airtight plugs. The device outlet was connected to a biomedical gas flow analyser (*CITREX H5, IMT Analytics AG., Switzerland*), while an adjustable aeraulic resistance was placed downstream to modulate outflow conditions. Tests were performed with the blower set at PWM duty cycles (DC%) ranging from 40 to 70%. For each DC%, the resistance was stepwise adjusted to control the flowrate (from free flow condition to zero flow with steps of approximately 20 L/min) and the value of the pressure at the FiBAC outflow section was recorded at each step.

The aeraulic characteristics of the PEEP valves were obtained delivering incremental air flows (range 0–150 L/min) to each isolated PEEP valve and measuring the air flowrate and pressure upstream from the valve.

Results of the experimental characterizations were used as input parameters for the LPM simulations.

### Lumped parameter model

**Fig. 2:**
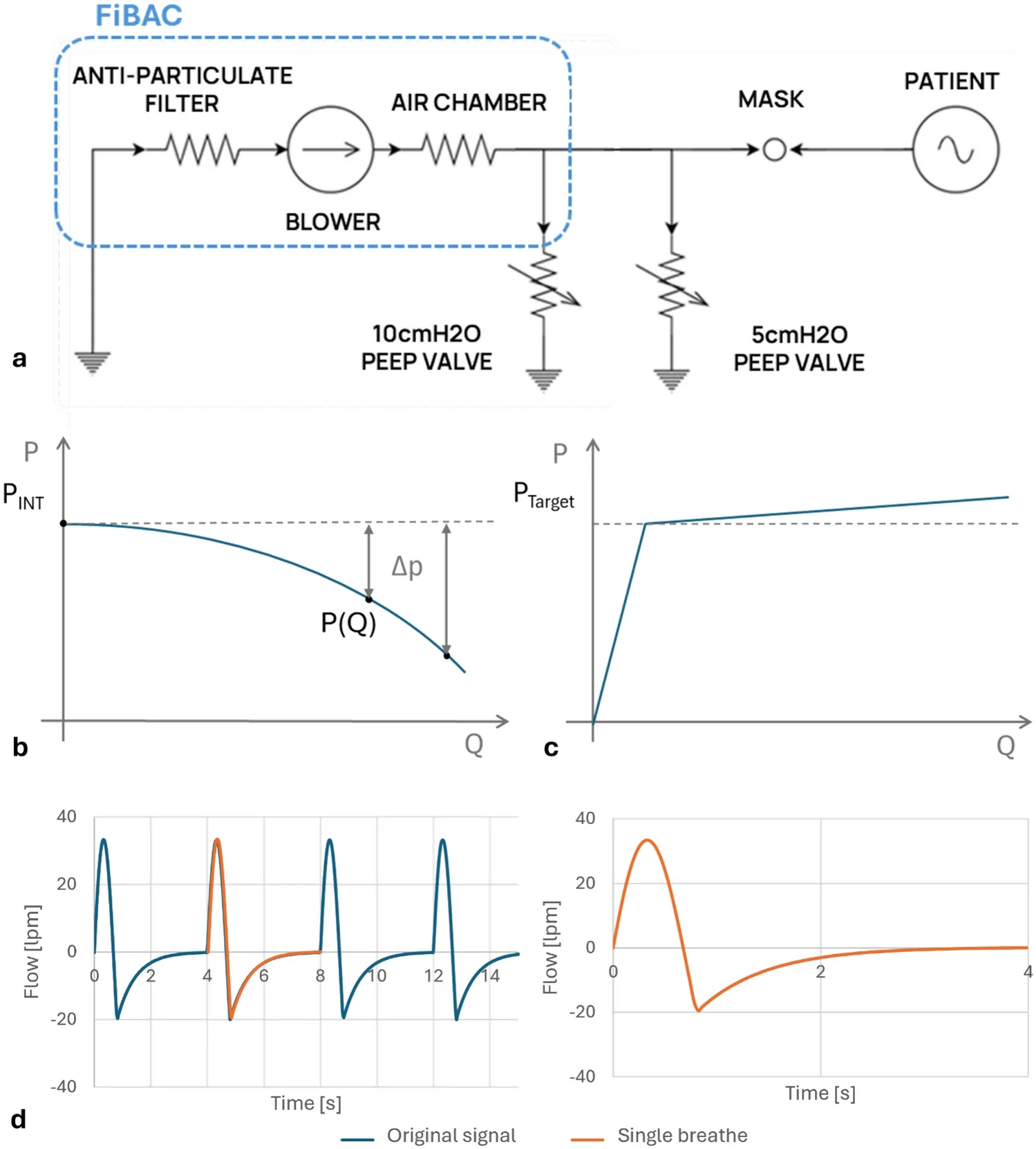
Scheme of the LPM topology (**a**); Paradigmatic characteristic curves of a centrifugal blower (**b**) and a PEEP valve (**c**); simulated patient breathing curves (**d**): airflow signal (left) and isolated single respiratory cycle (right) in a healthy subject during moderate-intensity exercise

A scheme of the LPM topology is shown in Fig. 2a. The model represents an ideal, leakage-free condition with FiBAC assembly modelled as a continuous, non-ideal pressure generator, the PEEP valves as variable resistive elements and the patient breathing as an oscillating flow source.

Model formulation was based on the conservation of air mass at the level of the oronasal mask-patient interface in the absence of any airflow leakage and considering air as an incompressible fluid, as follows:

1 $$ Q_{FiBAC} - Q_{PEEP5} - Q_{PEEP10} - Q_{PAT} = 0 $$

where Q_FiBAC_ is the flowrate generated by the device, Q_PEEP5_ and Q_PEEP10_ are the flowrates exiting the corresponding valves and Q_PAT_ is the flowrate at the patient airways (cycles of positive inspiration and negative expiration). The analytical representation of the pressure-flow characteristic of the FiBAC assembly was modelled assuming the behaviour of a standard centrifugal blower (shown in Fig. 2b): a downward sloping curve showing pressure decreasing from a maximum pressure value (P_INT_), as the flow increases from zero to maximum flow.

The FiBAC pressure-flow relation was expressed as a function of DC% and Q_FiBAC_ as follows:

2 $$ P\left( Q \right) = P_{int} \left( {DC} \right) - { }\Delta P\left( {\mathrm{Q}} \right) $$

3 $$ \left\{ {\begin{array}{*{20}l} {P_{int} = P_{{\left( {q = 0} \right)}} = a*DC\% - b} \\ {\Delta {\mathrm{P}} = {\mathrm{c}}_{1} {*}Q^{2} + c_{2} *Q} \\ \end{array} } \right. $$

where Q indicates Q_FiBAC_ for notation simplicity, P_INT_ represents the value of the pressure at zero flow rate (Q=0, the ordinate axis intercept in Fig. 2b) and ΔP is the difference between the pressure value at a given Q and the P_INT_. The value of P_INT_ was assumed to depend linearly on the DC% while the ΔP was modelled to have a quadratic dependence on the flowrate.

The variable resistance of the PEEP valves was modelled with a piecewise linear behaviour (Fig. 2c) consisting of an initial steep linear segment (P_1_(Q)) representing a high-resistance behaviour preceding the opening of the valve, followed by a second linear segment (P_2_(Q)) with a lower slope, representing a low-resistance behaviour in the open-valve flow range:

4 $$ \left\{ {\begin{array}{*{20}l} { P_{1} \left( Q \right) = d_{1} *Q } \\ {P_{2} \left( Q \right) = d_{2} *Q + e } \\ \end{array} } \right. $$

Patient spontaneous breathing was modelled using the flow profiles generated by an active lung simulator (ASL5000, Ingmar Medical), configured to replicate both physiological and pathological respiratory mechanics. The simulator was used to represent a healthy subject (at rest or at moderate/intense respiratory needs, for example at varying levels of physical exercise) as well as obstructive and restrictive respiratory conditions, namely i) chronic obstructive pulmonary disease (COPD), ii) acute respiratory distress syndrome (ARDS) and iii) asthma, each simulated at mild, moderate, and severe levels of severity. Table 1 reports the breathing rates related to all simulated patient conditions, according to library parameters of the ASL 5000 lung simulator [9].The flowrate–time signals (Fig. 2d) produced by the simulator were processed using a custom MATLAB (*MathWorks, R2025b, Natick, MA, USA*) script to isolate a representative single respiratory cycle; then, a spline interpolation was applied to obtain a smooth, continuous analytical representation of the patient’s breathing pattern. The root mean square error (RMSE) between the original signal and the spline interpolation has an average value of 1.23 L/min across all the spline-reconstructed curves.

**Table 1:** Software libraries breathing rate in different simulated scenarios at varying levels of severity (mild, moderate, severe)

Breathing rate (bpm)			
	Mild	Moderate	Severe
Adult healthy	8	15	25
Adult ARDS	25	30	35
Adult COPD	30	18	10
Adult ASTHMA	35	25	15

ARDS: Acute Respiratory Distress Syndrome; COPD: Chronic Obstructive Pulmonary Disease; bpm: breath per minute

The lumped-parameter model was simulated using the OpenModelica (*OMEdit, Open Source Modelica Consortium*) environment, and a dedicated code was developed. The model takes as input the spline interpolation coefficients representing the patient’s spontaneous breathing pattern, as previously obtained in MATLAB. The single respiratory cycle was extended in time through a dedicated periodicity function, allowing continuous simulation. The behaviour of each component was implemented in the code using the analytical expression of the relevant interpolation curves (Eqs. 2–4). The interaction between the FiBAC system, the PEEP5 and PEEP10 valves, and the patient at the mask-airway interface was modelled according to the mass conservation law expressed by *Eq. *1. Simulations were performed at three duty-cycle (DC) levels of the blower, namely 45, 55, and 65% of maximum power, corresponding to low-, medium-, and high-intensity operating conditions, respectively. This range was selected to identify, among a fixed, single-point operating setting for the blower, the most appropriate condition capable of ensuring the target PEEP across the widest possible range of simulated pathological conditions, as detailed in the Discussion.

### CFD simulation

**Fig. 3:**
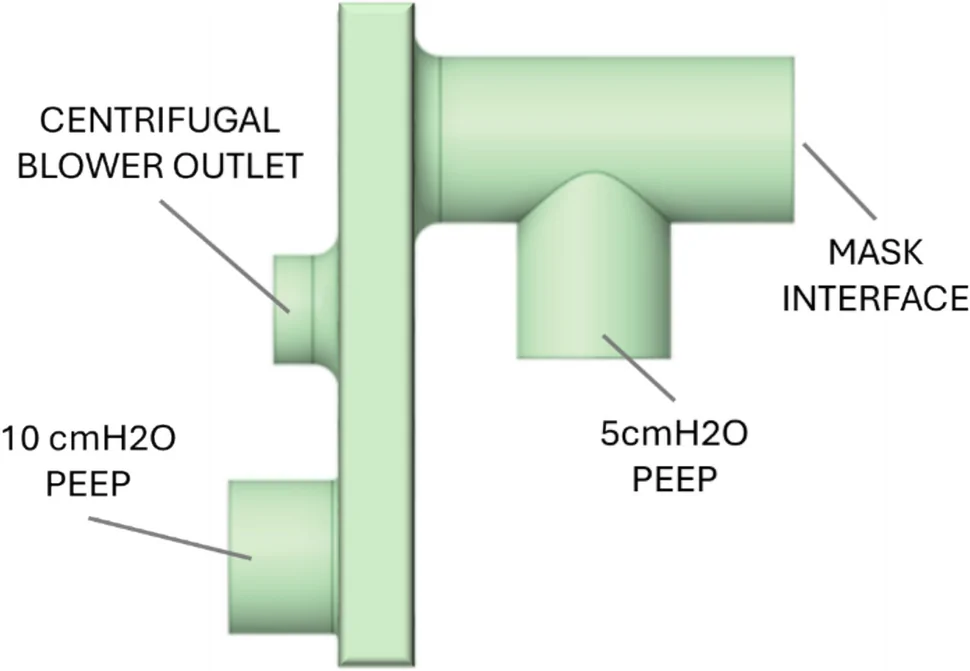
Overview of the passive-flow fluid three-dimensional computational domain of the CPAP device reconstructed to perform the CFD simulations

A three-dimensional computational fluid dynamics (CFD) model of the prototype was developed to numerically quantify the washout of the patient’s exhaled breath (clearance) from the device. Simulations were performed using ANSYS Fluent (*ANSYS, Inc., Canonsburg, PA, USA*) on the passive-flow fluid domain of the CPAP device, which is the fluid volume included within the centrifugal blower outlet, the air distribution chamber, the outlets of two PEEP valves, and the device-mask interface (Fig. 3).

After a mesh-sensitivity (grid-independence) analysis (data not shown), the computational domain was discretized using an unstructured tetrahedral mesh comprising ~1.3 million elements, including a five-layer near-wall inflation and an edge sizing of 6 × 10^−4^ m along the edges. The mesh was locally refined in regions with higher flow gradients, whereas areas with lower fluid-dynamic complexity were discretized with coarser elements.

Unsteady Reynolds-averaged Navier–Stokes simulations were performed using a pressure-based solver with the PISO algorithm for velocity–pressure coupling, second-order implicit temporal discretization, and second-order spatial discretization (second-order upwind scheme for the momentum equations and second-order scheme for pressure terms). Air was modelled as an incompressible Newtonian fluid with a density of 1.225 kg/m^3^ and a dynamic viscosity of 1.7894 × 10⁻^5^ kg/(m·s). The device was modelled as a non-deformable rigid body and the computational domain considered fully sealed, with no air leakage. Turbulence was modelled using the realizable k–ε model [12]. Under-relaxation factors for momentum and pressure were adjusted to improve numerical stability and convergence.

Values for the inlet and outlet flow-pressure boundary conditions were derived from the experimental characterizations of the PEEP valves, the centrifugal blower and the patient breathing profile.

Simulations were advanced using a time step of 0.004 s. The second-order upwind discrete scheme was adopted for the momentum terms and the second-order scheme for the pressure terms.

#### Volume washout

To evaluate adequate clearance of exhaled CO_2_ and no CO_2_ rebreathing, the washout of a passive scalar representing CO_2_ concentration was quantified under pure advection conditions [13]. In CFD, a passive scalar is a tracer transported by the airflow without influencing the flow field, pressure distribution, or fluid properties. Here, it was used as a surrogate marker of exhaled CO_2_ to estimate its clearance from the device volume.

At the onset of the expiration, (t = 0) the scalar was prescribed as 100% at the mask interface and set to zero elsewhere and at all the remaining boundaries. This approach enabled numerical estimation of the washout of the patient’s exhaled breath within the device. The residual fraction of the volume-averaged CO_2_ scalar within the functional device volume V was then computed as:

5 $$rCO_{2} \left( t \right) = \frac{1}{{V \cdot \left[ {CO_{2} } \right]_{0} }} \cdot \smallint \limits_{V} [CO_{2} ]\left( t \right) \cdot \partial V $$

where V is the functional volume of the device and t is the time elapsed since the beginning of expiration.

The convective transport of the passive scalar was discretized using a second-order scheme.

## Results

### Experimental characterization

**Fig. 4:**
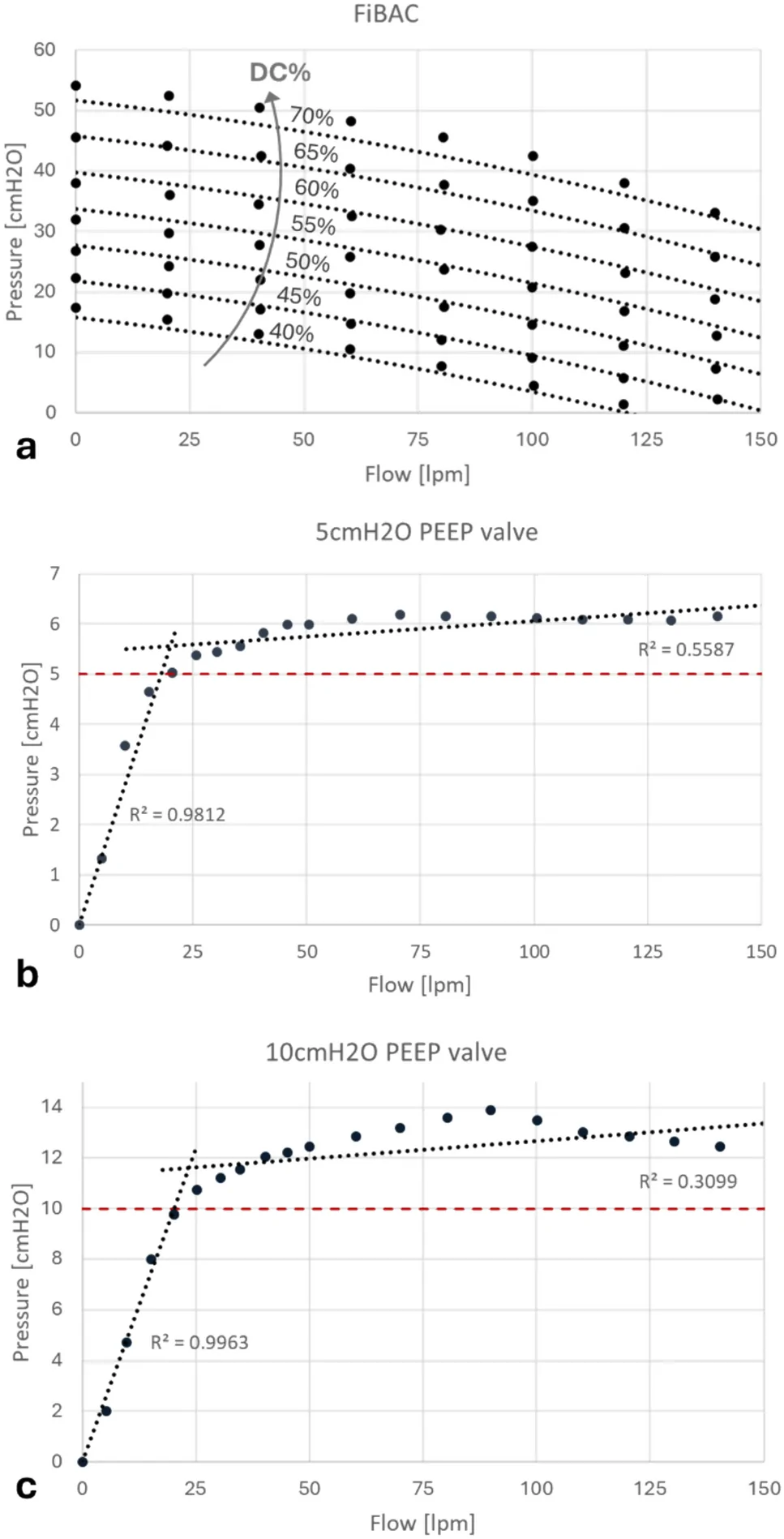
Pressure-flow curves obtained from the experimental characterization of each isolated circuit component, i.e., the FiBAC assembly (**a**), the 5cmH2O PEEP valve (**b**), and the 10cmH2O PEEP valve (**c**). Dots represent the experimental raw data, while dashed lines represent the analytical curves obtained by averaging the coefficients of the curves interpolating the experimental data

Figure 4a–c presents the pressure-flow plots resulting from the experimental characterization of the FiBAC assembly and the PEEP valves, along with the corresponding analytical curves obtained by averaging the coefficients of the interpolating curves fitted to the experimental data. It should be noted that these plots should be interpreted as the characterization of the isolated individual components, rather than as a comparison between the overall model and the assembled prototype.

Experimental data obtained from the characterization of the FiBAC assembly were used to estimate the coefficients required to construct the analytical curves defined in Eq. 3. The corresponding RMSE values ranged from 0.43 to 2.42 cmH_2_O across the tested duty-cycle percentages.

The PEEP valves’ characteristic plots, with their piecewise linear interpolation, led to the definition of the coefficients for Eq. 4. The difference between the experimental data and the resulting analytical curves exhibited root mean square errors (RMSE) of 0.28 cmH_2_O for the 5 cmH_2_O PEEP valve and 0.69 cmH_2_O for the 10 cmH_2_O valve. It is worth noting that the pressure-flow behavior of the PEEP valves (Fig. 4b–c) is non-ideal, exhibiting oscillations above their respective threshold values rather than an ideal, sharp cracking response.

### LPM simulations

**Fig. 5:**
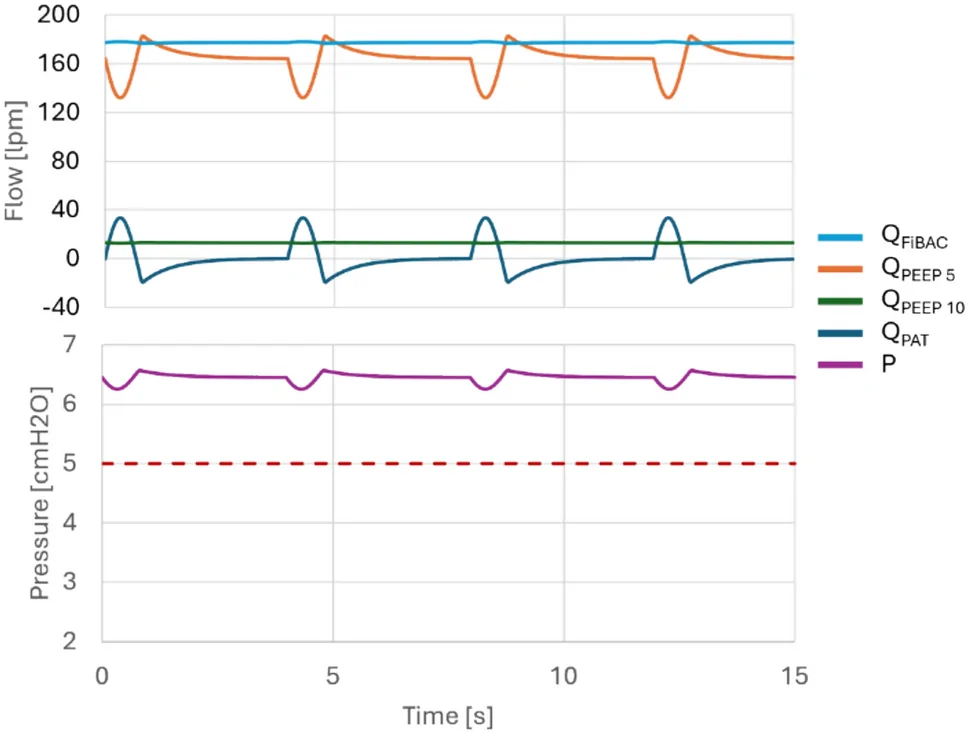
Time plots of LPM illustrating the case of a healthy subject breathing during moderate physical exercise with the device set at 55% DC. QFiBAC. QPEEP5, QPEEP10, QPAT are the flowrates exiting the FiBAC assembly, the 5 cmH_2_O PEEP valve, the 10 cmH_2_O PEEP valve and the simulated patient, respectively; P is the pressure at the airway openings

A representative graph from the LPM simulation is shown in Fig. 5, illustrating the case of a healthy subject breathing during moderate physical exercise with the device set at 55% DC.

The airflow exiting the PEEP valves and the FiBAC, as well as the pressure at the mask interface, were directly modulated by the patient’s breathing pattern. During inspiration, the patient draws flow from the system, thereby reducing the afterload on the FiBAC and shifting the blower operating point toward higher flow rates. This shift is associated with a decrease in pressure at the mask and a concomitant reduction in the PEEP valve opening, which leads to lower discharge of flow through the valves. In this phase, the PEEP5 valve is still opened and vents a substantial fraction of the blower-supplied flow to the atmosphere.

During expiration, the opposite occurs: the expiratory flow from the patient’s airways opposes the FiBAC-generated flow, increasing the system afterload and shifting the blower working point toward lower flow rates. Hence, at peak expiratory flow, the mask pressure reaches its maximum, and so the discharge through the PEEP valves, venting to the atmosphere both the patient’s exhaled air and the full blower-supplied flow.

During these dynamic phases, the PEEP10 valve is only mildly modulated, as the mask pressure remains predominantly within its high-resistance operating range. At the end-expiratory plateau, spontaneous breathing approaches a quasi-rest condition and the flow field evolves mainly within the device. The plateau pressure thus reflects the effective PEEP applied at the patient’s airway opening.

**Fig. 6:**
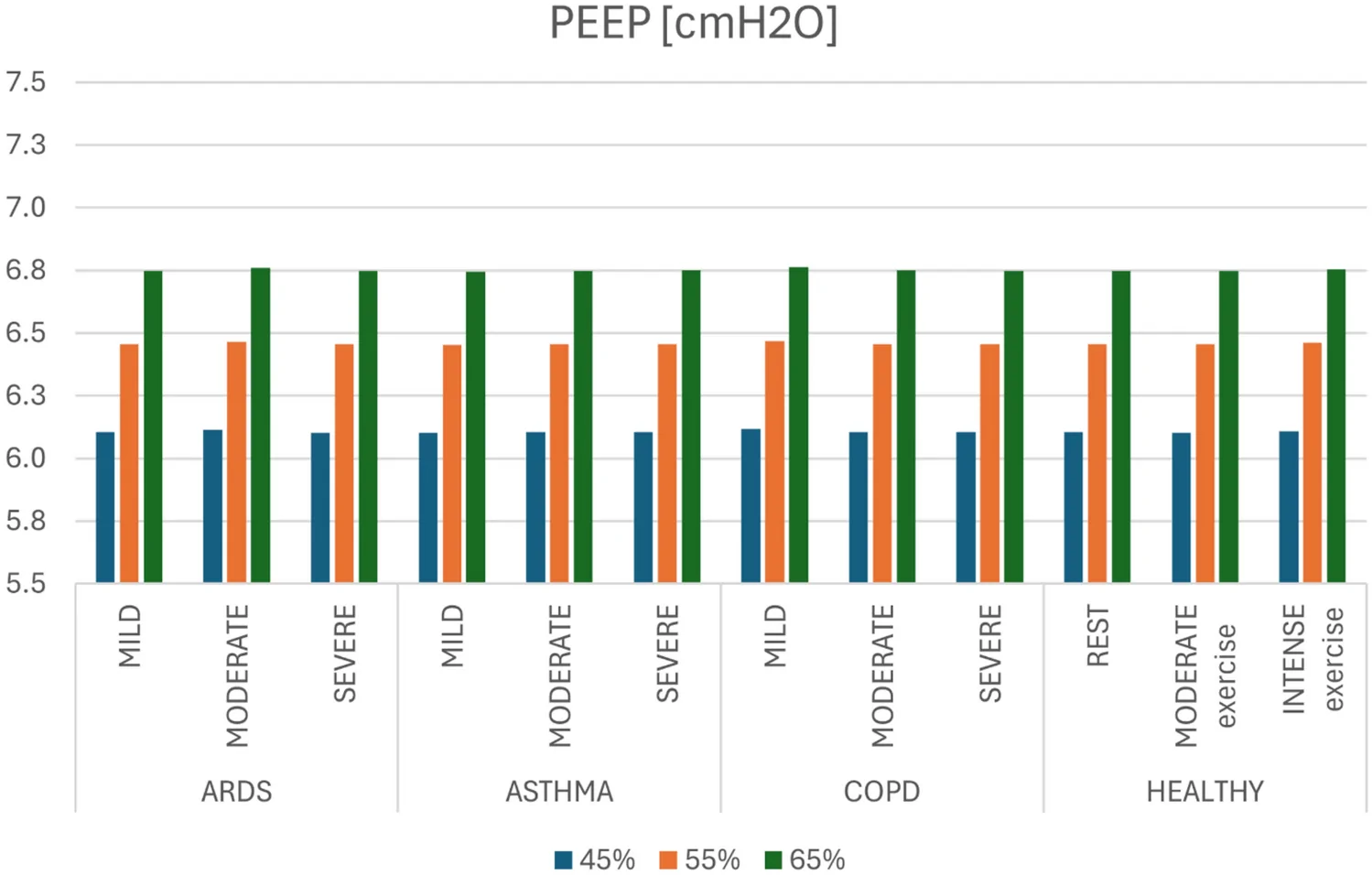
PEEP values represent deterministic outputs of the lumped-parameter model for each simulated respiratory profile (Healthy, ARDS, COPD, Asthma), with corresponding disease-severity levels (mild, moderate severe) or physical activity (rest, moderate or intense exercise), each simulated under three device operating conditions (45–55-65% of maximum power)

Figure 6 gathers the PEEP levels estimated across all the simulated scenarios, for each patient profile and simulated severity levels, under the three tested device operating conditions (DC: 45, 55 and 65%) representing low-, medium-, and high-intensity treatment settings, respectively.

PEEP consistently exceeded the target value of 5 cmH_2_O across all simulated scenarios and for all device settings (45, 55, and 65% duty cycle). The highest PEEP value was 6.76 cmH_2_O, recorded under the MILD COPD condition at 65% DC, whereas the lowest value was 6.10 cmH_2_O, in the MILD ASTHMA condition at 45% DC.

**Fig. 7:**
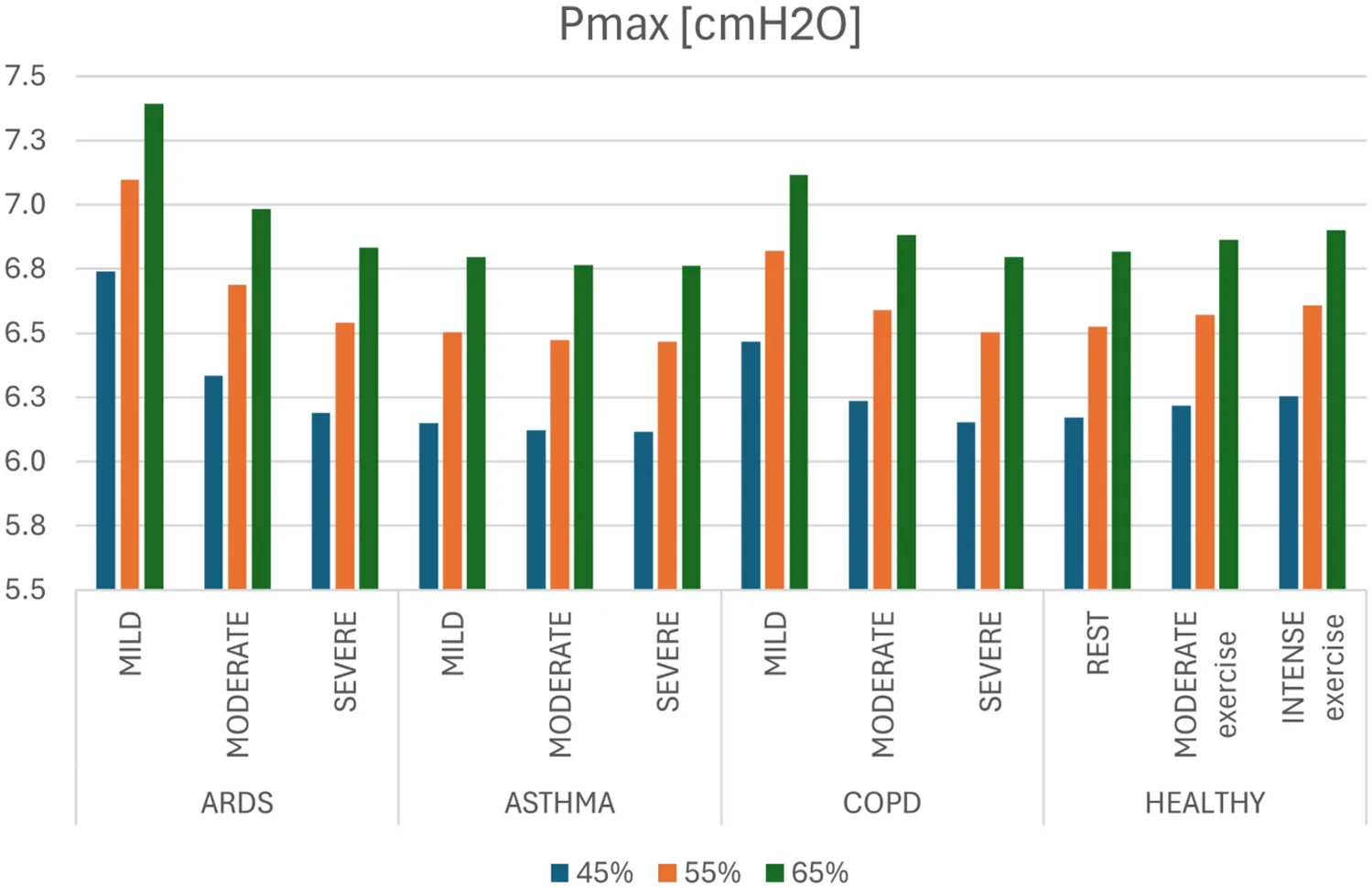
Maximum pressure values represent deterministic outputs of the lumped-parameter model for each simulated respiratory profile (Healthy, ARDS, COPD, Asthma), with corresponding disease-severity levels (mild, moderate severe) or physical activity (rest, moderate or intense exercise), each simulated under three device operating conditions (45–55-65% of maximum power)

Figure 7 show the values of maximum pressure as evaluated in the different simulated scenarios. The highest value of Pmax was 7.39 cmH_2_O measured under the MILD ARDS simulated profile with the device operating at 65% of maximum power.

Overall, our results show that PEEP increases with increasing device settings (DC %) and remains unaffected by disease severity or exercise intensity. In contrast, Pmax increases with DC % and decreases with disease severity. However, in the healthy subject, Pmax slightly increases with the respiratory rate.

### CFD simulations

**Fig. 8:**
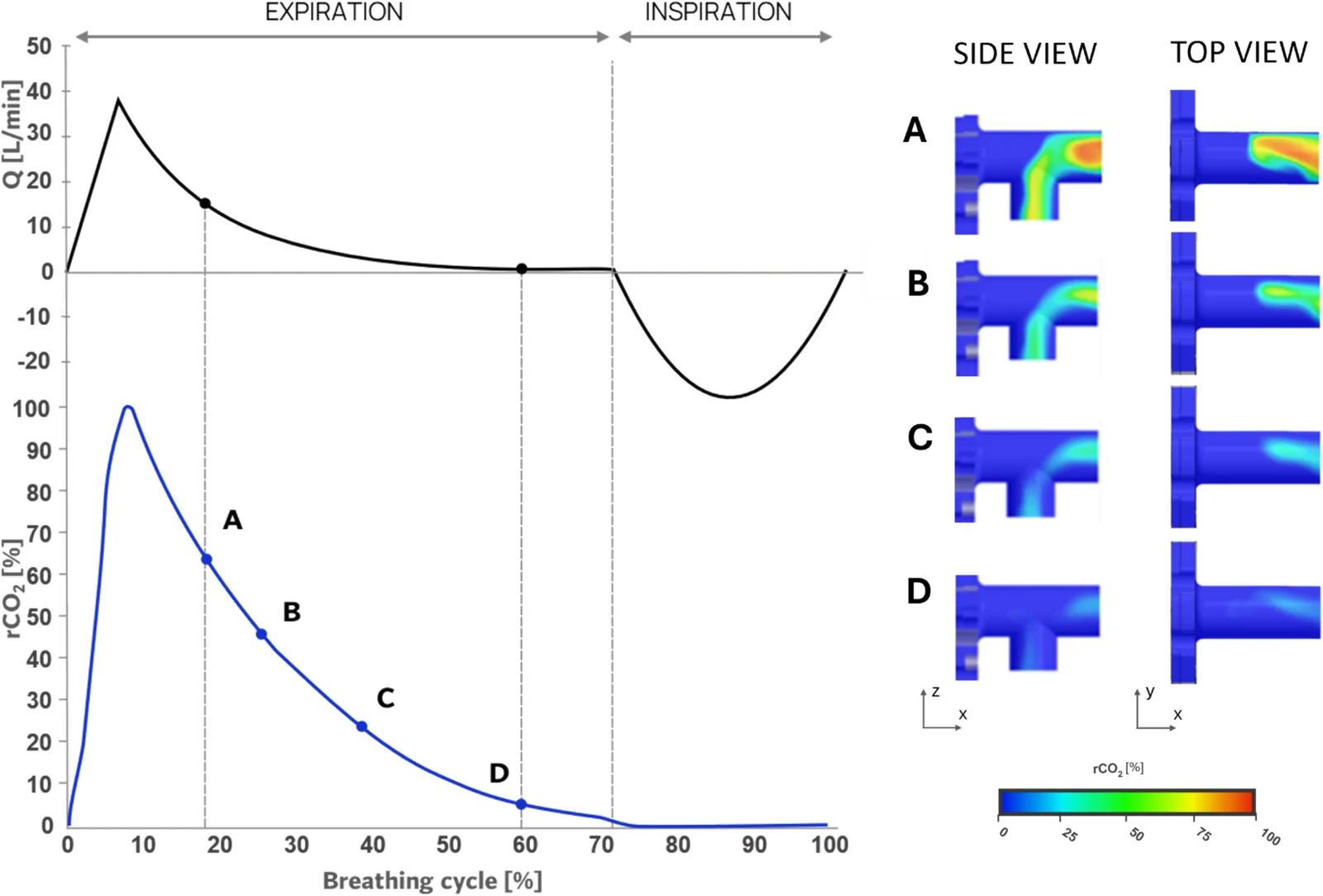
Left: Temporal evolution of the residual CO_2_ scalar (rCO_2_) over the breathing cycle. Right: Color maps showing volume distribution of rCO_2_ at selected time points

Figure 8 shows the residual CO_2_ scalar (rCO_2_) as a function of the normalized breathing cycle (percentage of breathing time), together with colour maps of the passive scalar concentration at selected time points. The rCO_2_ is reported for the second breathing cycle only, as the most representative period. The rCO_2_ follows a temporal trend that closely mirrors the expiratory flow profile, exhibiting an approximately negative-exponential decay, consistent with its strict dependence on the expiration flow velocity.

In detail, rCO_2_ decreased to 64% within 0.7s (25% of expiratory phase) and to 5% in 2.4s (86%) since the onset of expiration. At the beginning of the subsequent inspiration, only 2.3% of CO_2_-saturated exhaled air remained within the device.

## Discussion

In this study, we presented the results of a preclinical, integrated functional and safety characterization of a novel CPAP device designed and tailored for application in prehospital scenarios during ARF emergencies. The combined experimental characterization and computational modelling analysis we performed indicate that the adopted design solutions adequately meet both efficacy and safety requirements. Indeed, according to our results, the system achieves therapeutically effective airway pressures (target PEEP of 5 cmH_2_O) while maintaining intrinsic safety (PEEP < 10 cmH_2_O) and efficient clearance of exhaled gases.

In detail, our results:

(i) indicate a true potential of our device to maintain airway patency and alveolar recruitment when employed in real-world ARF scenarios, even under varying respiratory mechanics, which is particularly relevant because maintenance of adequate end-expiratory pressure is the primary determinant of CPAP efficacy in ARF patients [11, 14],
(ii) suggest that the device architecture intrinsically mitigates the risk of excessive airway pressurization and lung overdistension, as, in all simulated scenarios, peak pressure values remained consistently below predefined safety limits [15, 16], and
(iii) highlight no risk for cycle-to-cycle CO_2_ rebreathing.

In this regard, the proposed device is not intended to provide pressure support or to guarantee a target tidal volume. Patients with severe hypercapnic ARF and respiratory muscle pump exhaustion may require bilevel NIV or pressure-support ventilation to improve alveolar ventilation and should therefore undergo prompt expert assessment and escalation of care. The present system is instead intended as a low-level, temporary CPAP support for early out-of-hospital use.

This balance between efficacy and safety supports the appropriateness of the design rationale. This aspect is particularly relevant when considering the heterogeneous pathophysiology of ARF. In hypoxemic ARF, CPAP may be beneficial by increasing end-expiratory lung volume, promoting alveolar recruitment, and improving oxygenation. In contrast, in hypercapnic ARF, particularly during severe acute exacerbations of COPD, the selection of PEEP requires caution, as excessive PEEP may worsen dynamic hyperinflation, increase expiratory load, and potentially contribute to rapid clinical deterioration. Accordingly, the present device was intentionally designed to deliver a conservative, fixed, low-level PEEP of approximately 5 cmH_2_O, rather than high or adjustable CPAP levels. The 10 cmH_2_O valve works as an additional safety-limiting component against overpressurization, and is not an intended therapeutic pressure level. These considerations are especially important for use by first responders or potentially untrained bystanders, who should not apply high CPAP levels in patients with suspected severe acute exacerbation of COPD and should instead use the device only as an early temporary bridge while awaiting expert medical assessment.

Of note, device safety was established as the primary design constraint from the earliest design stage and addressed by selecting a centrifugal blower as the device actuation system, owing to its intrinsic safety characteristics and limited pressure-generation capability. The integration of two mechanical passive PEEP valves to provide pressure regulation further enhanced the device safety. The dual-valve configuration plays indeed a central role in both the device’s efficacy and safety logic, with built-in redundancy. Specifically, the PEEP5 valve primarily acts as a pressure stabilizer, operating mostly within its low-resistance regime and thereby preventing excessive pressure excursions. It functions as a dynamic airflow discharge pathway, venting excess flow to properly set the desired target pressure during transient phases of the respiratory cycle. In turn, during normal operating conditions (i.e., no malfunctioning), the PEEP10 constantly operates below its opening pressure, thereby acting as a second safety element that intervenes only in the case of unexpected excessive pressure load. In normal operating conditions, the PEEP10 is constantly within its high-resistance regime. Consequently, this element introduces a leakage into the system’s airflow dynamics; nevertheless, airflow plots clearly show that such leakage is so small as to not relevantly affect the expected device operation.

To maximize the efficacy of this passive pressure regulation module, we opted for the use of fixed-point PEEP valves instead of adjustable valves. This choice was motivated by several factors: first, fixed-point PEEP valves have higher precision compared with adjustable valves [17]; second, they enhance broader applicability, and, third, safety, as the operator cannot alter the preset values. Accordingly, fixed-point valves ensure greater operational repeatability and improved compliance with the intended design specifications. This design choice differentiates the proposed system from currently available pre-hospital CPAP devices and might allow to overcome the operational constraints of existing pre-hospital CPAP systems, such as the Boussignac CPAP. Those systems, although clinically useful in equipped pre-hospital settings, do not address the specific use case targeted by the present device, namely a compact, fully standalone, battery-powered CPAP device requiring no external oxygen or pneumatic source and no manual setting of ventilatory parameters.

The analysis of the functional device characteristics (performance) confirmed the expected dependence of pressure generation on duty-cycle (DC %) settings of the centrifugal blower, in which the pressure–flow relationship is indeed governed by the operating point along the characteristic curve of the blower. The observed monotonic increase in pressure with increasing DC % showed only minor variation across simulated conditions, confirming the internal mechanical coherence of the model and indicating that the selected operating range allows predictable modulation of support intensity.

Importantly, the evidence that the device power setting is not critical to achieve the target therapeutic PEEP level opens a wide range of choice for it, supporting at the same time the choice of adopting a constant setting for it. This insight was enabled by the use of a lumped-parameter model, in which the device components’ behaviour was represented through parametric analytical expressions, thereby allowing rapid and automated assessment of the overall device performance across a wide range of device operating settings and scenarios, including different device operating levels (DC %) combined with varying simulated patient breathing profiles. Importantly, the parameters for the LPM elements were obtained from the experimental characterization of the actual prototype components, which ensured robustness and improved fidelity of the LPM results. In this work, the LPM had the role of driving us through the design process; in this view, model simplicity is for sure a limitation, but it is also a key advantage, as it removes the confounding factors arising from the subject's complex clinical condition, allowing the designer to focus on the main characteristics of the device: effectiveness and safety. Our modelling and testing approach ultimately allowed identification of the most appropriate operating condition for the device, that is DC = 55%. One early chosen design feature was indeed that of having a fixed, single-point, operating setting for the blower, such that no operator pre-setting manoeuvres are needed prior to apply the device. This implies increasing usage simplicity, working repeatability, and safety against mis-manoeuvres. However, the decision of such single operating point must satisfy the criterion of making the device suitable for the widest possible range of interesting pathological conditions. Simulation results showed that the target PEEP (5 cmH_2_O) could even be achieved, under ideal conditions, at lower power levels (e.g., DC = 45%). Nonetheless, real-world operation is expected to deviate from simulated behaviour. Therefore, a more conservative operating point was selected, to ensure robust and reliable performance under practical clinical conditions. Consequently, we selected an operating setting corresponding to 55% of the maximum power as the preferred configuration. This choice was informed by the results of the simulation analysis we performed as well as by additional converging considerations: (i) optimization of battery life, which may be critical in emergency-use scenarios; (ii) the incorporation of a safety margin to account for occasional deviations of the device operation from the nominal operating conditions (e.g., power fluctuations, reduced battery capacity); and (iii) limitation of the influence of possible variable degree of mask fitting and air leakage at the mask–patient face interface due to potential misapplication or inter-individual anatomical variability.

Although simplified, the proposed LPM allowed the dynamic interaction between the device, the PEEP valves and the simulated breathing profile to be described. It should be emphasized that the LPM was not intended to reproduce the full pathophysiological response of the human respiratory system in different disease states. Obstructive sleep apnea, asthma, COPD, ARDS, and other causes of ARF involve distinct mechanisms, including upper-airway collapse, bronchial narrowing, increased airway resistance, reduced lung compliance, alveolar derecruitment, and impaired gas exchange. These mechanisms cannot be comprehensively represented by a simple lumped-parameter model. In the present study, the disease conditions were therefore not modelled through the PEEP valves, which only represented the pressure-regulating components of the device, but through representative breathing profiles generated by an active lung simulator configured to reproduce physiological and pathological respiratory mechanics.

As mentioned, to select the most appropriate device operating point, the device performance was evaluated—for each tested DC % setting—across different breathing conditions with increasing levels of disease severity. The observed stepwise reduction in the maximum delivered pressure as disease severity increases reflects effective fluid-dynamic coupling between patient respiratory conditions and device output, which is consistent with expected pathophysiology of respiratory support in ARF: as respiratory mechanics deteriorate, the simulated patient imposes greater instantaneous flow requirements on the system, shifting the operating point of the blower toward regions of lower pressure generation. From a design perspective, this is particularly relevant because it confirms passive device self-adaptation: the system does not deliver indeed a “static” pressure level; rather, it entirely passively adapts its operation to different respiratory patterns—continuously throughout the respiratory cycle—as a result of the intrinsic flow-pressure characteristics of the blower and its fluid–dynamic interaction with the patient, without the need for complex sensing, active feedback control systems, or operator-dependent parameter tuning. This observed behaviour further supports the robustness and translational validity of the proposed solution in prehospital emergency settings, where simplicity, rapid employment, and consistency over user variability are critical requirements.

The proposed LPM allowed the dynamic interaction between the device, the PEEP valves, and the simulated breathing profile to be described, that is, to assess system-level pressure–flow interactions and to estimate global performance variables, such as PEEP and maximum airway-opening pressure. On the other hand, the model was not able to resolve local three-dimensional fluid-dynamic phenomena, including turbulence, flow separation, recirculation, local mixing, or spatial pressure and velocity gradients. These geometry-dependent aspects were therefore investigated separately through CFD analysis, which complemented the LPM by providing spatial information on airflow distribution and exhaled CO_2_ clearance within the device.

As far as the risk for CO_2_ rebreathing is concerned, the results of the CFD simulations (washout analysis) indicate a low potential risk for stagnation of exhaled air inside the airflow channel, thereby suggesting that cycle-to-cycle CO_2_ accumulation in the mask is unlikely and that the system architecture enables effective clearance of exhaled gas. The residual CO_2_ scalar exhibited an approximately exponential decay over expiration, with nearly complete CO_2_ washout achieved by the end of the expiratory phase, with only a very small residual fraction (2.3%) still present at the onset of the subsequent inspiration. From a clinical perspective, the risk of hypercapnia due to rebreathing appears therefore negligible under the simulated conditions.

Collectively, our findings suggest that the device architecture is compliant with the operational constraints of out-of-hospital respiratory support in emergency environments. The adoption of fixed mechanical PEEP valves was an intentional design choice, rather than a limitation of the proposed architecture. Indeed, although in conventional hospital-based CPAP systems, adjustable pressure settings may be advantageous to tailor respiratory support to specific diseases and individual patient needs, the present device was conceived for early, temporary, out-of-hospital application, potentially by first responders or laypersons, in emergency scenarios in which the underlying diagnosis is often unknown and no trained operator may be available to select or adjust ventilatory parameters. In this setting, variable valves or adjustable PEEP levels would increase operational complexity and could expose patients to the risk of inappropriate settings. For this reason, a fixed target PEEP of 5 cmH_2_O was selected as a trade-off between therapeutic benefit and safety, providing a degree of alveolar recruitment and ventilatory support while minimizing the risk of excessive lung distension or barotrauma. An additional 10 cmH_2_O valve was included as a safety-limiting component to prevent potentially harmful pressure levels. Thus, the fixed-valve configuration is consistent with the intended role of the device as a simple, ready-to-use bridge to advanced hospital-based intervention, rather than as a fully adjustable ventilatory platform. While conventional CPAP systems typically require pre-setting and active monitoring to adjust operational parameters during treatment, that is, a certain level of expertise, the simplified architecture of our device minimizes operator dependence on medical/technical expertise while preserving therapeutic effectiveness and safety. In particular, by eliminating the need for manual parameter tuning and complex interfaces, the system may overcome those barriers that have historically limited the widespread adoption of CPAP in emergency ARF scenarios. In this context, the proposed system has the potential to effectively shorten time-to-support in ARF, which is a recognized factor influencing patient outcomes [18–20]. Our device might indeed enable prompt initiation of non-invasive respiratory support even outside clinical environments by personnel with no technical training, including first responders. These characteristics extend its potential applicability across a wide range of real-world scenarios where simplicity, portability, and robustness are essential, including ambulance transport or remote/resource-limited settings, and emergency first-response kits deployed throughout the territory, similarly to public-access AED programmes.

This device could therefore be applied in multiple out-of-hospital scenarios including (i) home, work, public places or street emergencies by lay bystanders; (ii) nursing homes, retirement homes, elder care facilities; (iii) ambulances not equipped with or do not have the expertise or time to initiate non-invasive ventilation; (iv) non-intensive hospital wards that do not have promptly available non-invasive ventilation devices or expertise, while waiting for the arrival of the medical emergency team. Its intended use is for early, temporary support in patients presenting with acute respiratory distress, particularly in the initial out-of-hospital phase in which the underlying diagnosis may still be uncertain and treatment delays may contribute to clinical deterioration. Accordingly, the proposed device is not intended to replace diagnostic assessment, provide disease-specific ventilatory management i.e., clinician-guided NIV, or advanced ventilatory management, but rather to enable rapid initiation of low-level CPAP support while awaiting expert evaluation and escalation of care when needed.

These considerations are further supported by a recent study from our group where we assessed the device usability under more realistic conditions, with the device applied by multiple users to a manikin connected to an active lung simulator [9]. A comparison of the PEEP values delivered by the device in different simulated pathological respiratory scenarios relevant to ARF showed a difference of 1.1–1.3 cmH_2_O between the computed (this study) and the experimental (previous work) data. These results validate the reliability of the developed model and demonstrate its ability to predict, with significant accuracy, the real-world behaviour of the device.

### Study strengths and limitations

A major strength of this study is the integrated, multi-methodological testing framework that combine (i) experimental characterization, (ii) lumped-parameter modelling, and (iii) CFD simulations. This approach allowed to evaluate the device performance across complementary scales, from component-level behaviour to system-level dynamics. In particular, the use of experimentally derived parameters ensured physiologically and mechanically realistic LPM inputs. Likewise, boundary conditions of the numerical model were also grounded in measured data rather than assumptions. Finally, different simulations spanning multiple respiratory phenotypes broadened the relevance of the analysis in the perspective of real-world use cases. Overall, this integrated framework increases confidence in the robustness of the device performance assessment.

Several limitations should, however, be acknowledged. First, the characteristic curves of the PEEP valves were interpolated using a piecewise linear behaviour, although the actual valve characterization shows a certain degree of non-linearity, especially in the range of high flowrates. Even if the interpolation error was proven quite limited, one should be aware that the model provides an idealized representation of PEEP valve functional characteristics, that may deviate from the real operating conditions.

Second, the modelling framework assumed leakage-free conditions, whereas several potential sources of air leakage may occur in real-world use (bad components connections, mask mispositioning or any other improper use of the device). To account for potential air leakage, we selected the device operating point with an appropriate safety margin. In this regard, preliminary LPM simulations exploring the effect of increasing leakage flow (0–120 lpm) at different blower duty-cycle settings (45, 55, and 65%) showed that a 55% duty cycle was sufficient to maintain the target PEEP level even under simulated leakage conditions, supporting the choice of this operating point (see Supplementary Material, Fig. S1). In this context, LPM simulations were of great help as a decision-making support during the design phase. In real-world use, however, we acknowledge that air leakage may be rather uncontrollable and unpredictable, and the present leakage-free assumption does not allow us to establish whether the intended CPAP pressure would still be reliably maintained under such conditions; this aspect will need to be specifically addressed in future work, for instance through dedicated bench testing with controlled leak simulation. Air leakages might also influence CO_2_ washout and rebreathing: on the one hand, air leakage towards the atmosphere might be an additional—beneficial—CO_2_ venting pathway; on the other hand, leakage may reduce the effective airflow within the device, contributing in creating CO_2_ stagnation/recirculation regions, thereby increasing the time required to eliminate exhaled CO_2_. Although our results (2.3% of residual CO_2_ at the end of expiratory phase) may suggest that these effects are unlikely to pose a significant risk for the patient, this potential issue certainly deserves further investigations.

Third, our modelling analysis relied on simulated patient breathing profiles rather than in vivo measurements, which may not fully capture real-world variability and patient heterogeneity. The breathing rates and respiratory profiles used in the simulations were derived from the ASL5000 software libraries and were selected to provide standardized and reproducible testing conditions. Therefore, they may not cover the full spectrum of real-world ARF presentations, including severe acute exacerbations of COPD (e.g., rapid shallow breathing and high respiratory rates). Anatomical variability of the upper airway and interface fit was not indeed represented, and airway resistances were simplified for modelling purposes. Furthermore, respiratory parameters were kept constant within each simulated scenario, which may instead vary during in vivo device application. In addition, the LPM should be interpreted as a preliminary design-assessment tool, rather than as a comprehensive model of lung mechanics or disease-specific respiratory response. In particular, the LPM was not intended to reproduce the full complexity of patient–device interaction or the physiological response of the human respiratory system under normal and pathological conditions. Instead, the LPM was conceived as an engineering model for early design assessment, aimed at describing system-level pressure–flow interactions between the device components and representative breathing profiles. More realistic preclinical validation belongs to successive bench-test activities carried out with the final device prototype. This aspect might also limit the general validity of the results of the CFD analysis, as possible changes in respiratory rate, tidal volume, expiratory flow, mask fit and leakage were not considered. Longer-duration, multi-cycle simulations accounting for variable breathing patterns and patient–interface conditions are therefore required to exclude cumulative CO_2_ retention under more realistic operating scenarios.

While these assumptions enabled controlled and interpretable evaluation of the results, real-world variability may influence device performance and should be investigated in future studies incorporating air leaks and interface fit variability, and dynamically varying respiratory mechanics within the same experiment.

Lastly, human-factor aspects, such as patient’s comfort and tolerability, were not assessed in this study. Given these idealized experimental and computational assumptions, conclusions regarding the physiological validity and expected clinical performance of the device should accordingly be regarded as preliminary. Pilot clinical studies are therefore needed to confirm safety, effectiveness, and usability in real-world emergency settings.

## Conclusions

This study presents the integrated functional and safety characterization of a novel CPAP device specifically designed for immediate deployment in out-of-hospital respiratory emergencies. Combined experimental measurements, lumped-parameter modelling, and CFD simulations suggested that the system can deliver therapeutically effective airway pressures while maintaining intrinsic safety through passive mechanical pressure regulation. The device showed stable pressure generation across different simulated pathological respiratory conditions relevant to ARF, and efficient clearance of exhaled gas, indicating a low potential for CO_2_ rebreathing. These findings support the technical robustness and physiological validity of our system, within the idealized conditions under which the simulations were performed, in particular the assumption of periodic, predictable breathing cycles and leak-free operation; further work will be needed to assess the device's behaviour under the more irregular, effort-dependent breathing patterns and mask leakage typically encountered in clinical practice. Overall, these results highlight the system's potential for safe and effective respiratory support in the prehospital setting, and clinical studies are ongoing to validate our results and confirm the device actual translational impact in scenarios where expert medical support is not available.

## Supplementary Information

Below is the link to the electronic supplementary material.

Supplementary file1 (DOCX 24 KB)
