# Supplementary material for: Design assessment and preliminary performance evaluation of a novel device for non-invasive emergency treatment of acute respiratory failure
Source: Biomed Eng Lett. 2026 Oct 25;16(6):1607–20. doi: 10.1007/s13534-026-00619-3 (PMC13740428; doi:10.1007/s13534-026-00619-3)
Supplement: Supplementary file 1 [file 13534_2026_619_MOESM1_ESM.docx]

Journal: Biomedical Engineering Letters

*Design Assessment and Preliminary Performance Evaluation of a Novel Device for Non-Invasive Emergency Treatment of Acute Respiratory Failure*

*Federica Ciceri Negri^1†^, Eleonora Salurso^1†^, Giuseppe Giardina^2^, Filippo Consolo^3^, Giovanni Landoni^2,3^, Alberto Cesare Luigi Redaelli^1*^, Gianfranco Beniamino Fiore^1^*

^1^Department of Electronics, information and bioengineering, Politecnico di Milano, Milan, Italy

^2^Department of Anaesthesia and Intensive Care, IRCCS San Raffaele Scientific Institute, Milan, Italy

^3^School of Medicine, Vita-salute San Raffaele University, Milan, Italy

† These authors contributed equally.

* Corresponding author: Alberto Redaelli, [alberto.redaelli@polimi.it](mailto:alberto.redaelli@polimi.it)

**Supplementary – leakage test**

As acknowledged in the Limitations section of the manuscript, the lumped-parameter model (LPM) presented in the main text was developed under leak-free conditions. To provide preliminary insight into the device's behaviour in the presence of leakage, additional simulations were performed exploring how increasing leakage flow affects the ability of the system to maintain the target PEEP level.

**Methods**
Simulations were performed with the patient flow component set to zero (Qpatient = 0 lpm), in order to isolate the effect of leakage on PEEP maintenance without the confounding contribution of breathing-induced flow variations. Leakage was simulated as an additional airflow escaping from the circuit at the mask level. Leakage flow (Qleakage) was in the arbitrary range from 0 to 120 lpm. Simulations with leakage were repeated for three blower duty-cycle (DC%) settings — 45%, 55%, and 65% — corresponding to the range of operating conditions explored in the main manuscript.

**Results**
The results show that PEEP tends to drop once leakage flow exceeds a certain threshold, and that this threshold depends on the duty-cycle setting of the device. At a DC% of 45%, PEEP begins to decline at a leakage flow of approximately 90 lpm. In contrast, at DC% settings of 55% and 65%, PEEP remains stable and above the target level (5 cmH2O) throughout the entire tested range, up to 120 lpm.

PEEP (cmH2O)

| Leakage Flow (lpm) | DC% = 45% |
| --- | --- |
| 0 | 6.10382 |
| 4 | 6.08028 |
| 8 | 6.05674 |
| 12 | 6.0332 |
| 16 | 6.00966 |
| 20 | 5.98612 |
| 24 | 5.96258 |
| 28 | 5.93904 |
| 32 | 5.91549 |
| 36 | 5.89195 |
| 40 | 5.86841 |
| 44 | 5.84486 |
| 48 | 5.82132 |
| 52 | 5.79777 |
| 56 | 5.77423 |
| 60 | 5.75068 |
| 64 | 5.72713 |
| 68 | 5.70359 |
| 72 | 5.68004 |
| 76 | 5.65649 |
| 80 | 5.63294 |
| 84 | 5.6094 |
| 88 | 5.58585 |
| 91.2217 | 5.56688 |
| 91.2217 | 5.56911 |
| 92 | 5.49932 |
| 96 | 5.13972 |
| 100 | 4.7786 |
| 104 | 4.41599 |
| 108 | 4.0519 |
| 112 | 3.68636 |
| 116 | 3.31937 |
| 120 | 2.95096 |
| 124 | 2.58115 |
| 128 | 2.20995 |
| 132 | 1.83738 |
| 136 | 1.46345 |
| 140 | 1.08819 |
| 144 | 0.711604 |
| 148 | 0.333711 |
| 152 | -0.0454736 |
| 156 | -0.425935 |
| 160 | -0.80766 |
| 164 | -1.19063 |
| 168 | -1.57484 |
| 172 | -1.96026 |
| 176 | -2.3469 |
| 180 | -2.73472 |
| 184 | -3.12373 |
| 188 | -3.5139 |
| 192 | -3.90523 |
| 196 | -4.2977 |
| 200 | -4.6913 |
| 200 | -4.6913 |

| Leakage Flow (lpm) | DC% = 55% |
| --- | --- |
| 0 | 6.45572 |
| 4 | 6.43202 |
| 8 | 6.40832 |
| 12 | 6.38462 |
| 16 | 6.36092 |
| 20 | 6.33722 |
| 24 | 6.31352 |
| 28 | 6.28982 |
| 32 | 6.26612 |
| 36 | 6.24242 |
| 40 | 6.21872 |
| 44 | 6.19502 |
| 48 | 6.17132 |
| 52 | 6.14762 |
| 56 | 6.12392 |
| 60 | 6.10021 |
| 64 | 6.07651 |
| 68 | 6.05281 |
| 72 | 6.02911 |
| 76 | 6.0054 |
| 80 | 5.9817 |
| 84 | 5.958 |
| 88 | 5.93429 |
| 92 | 5.91059 |
| 96 | 5.88689 |
| 100 | 5.86318 |
| 104 | 5.83948 |
| 108 | 5.81577 |
| 112 | 5.79207 |
| 116 | 5.76836 |
| 120 | 5.74466 |
| 124 | 5.72095 |
| 128 | 5.69725 |
| 132 | 5.67354 |
| 136 | 5.64984 |
| 140 | 5.62613 |
| 144 | 5.60242 |
| 148 | 5.57872 |
| 149.997 | 5.56688 |
| 149.997 | 5.56936 |
| 152 | 5.36947 |
| 156 | 4.9695 |
| 160 | 4.56848 |
| 164 | 4.16642 |
| 168 | 3.76332 |
| 172 | 3.35921 |
| 176 | 2.95408 |
| 180 | 2.54795 |
| 184 | 2.14083 |
| 188 | 1.73273 |
| 192 | 1.32365 |
| 196 | 0.913616 |
| 200 | 0.502624 |
| 200 | 0.502624 |

| Leakage Flow (lpm) | DC% = 65% |
| --- | --- |
| 0 | 6.74862 |
| 4 | 6.72483 |
| 8 | 6.70104 |
| 12 | 6.67725 |
| 16 | 6.65346 |
| 20 | 6.62967 |
| 24 | 6.60588 |
| 28 | 6.58209 |
| 32 | 6.5583 |
| 36 | 6.53451 |
| 40 | 6.51072 |
| 44 | 6.48693 |
| 48 | 6.46314 |
| 52 | 6.43935 |
| 56 | 6.41555 |
| 60 | 6.39176 |
| 64 | 6.36797 |
| 68 | 6.34418 |
| 72 | 6.32039 |
| 76 | 6.29659 |
| 80 | 6.2728 |
| 84 | 6.24901 |
| 88 | 6.22521 |
| 92 | 6.20142 |
| 96 | 6.17763 |
| 100 | 6.15384 |
| 104 | 6.13004 |
| 108 | 6.10625 |
| 112 | 6.08245 |
| 116 | 6.05866 |
| 120 | 6.03487 |
| 124 | 6.01107 |
| 128 | 5.98728 |
| 132 | 5.96348 |
| 136 | 5.93969 |
| 140 | 5.91589 |
| 144 | 5.8921 |
| 148 | 5.8683 |
| 152 | 5.84451 |
| 156 | 5.82071 |
| 160 | 5.79692 |
| 164 | 5.77312 |
| 168 | 5.74933 |
| 172 | 5.72553 |
| 176 | 5.70174 |
| 180 | 5.67794 |
| 184 | 5.65414 |
| 188 | 5.63035 |
| 192 | 5.60655 |
| 196 | 5.58275 |
| 198.668 | 5.56688 |
| 198.668 | 5.56953 |
| 200 | 5.42765 |
| 200 | 5.42765 |

| Leakage Flow (lpm) | target |
| --- | --- |
| 0 | 5 |
| 4 | 5 |
| 8 | 5 |
| 12 | 5 |
| 16 | 5 |
| 20 | 5 |
| 24 | 5 |
| 28 | 5 |
| 32 | 5 |
| 36 | 5 |
| 40 | 5 |
| 44 | 5 |
| 48 | 5 |
| 52 | 5 |
| 56 | 5 |
| 60 | 5 |
| 64 | 5 |
| 68 | 5 |
| 72 | 5 |
| 76 | 5 |
| 80 | 5 |
| 84 | 5 |
| 88 | 5 |
| 92 | 5 |
| 96 | 5 |
| 100 | 5 |
| 104 | 5 |
| 108 | 5 |
| 112 | 5 |
| 116 | 5 |
| 120 | 5 |
| 124 | 5 |
| 128 | 5 |
| 132 | 5 |
| 136 | 5 |
| 140 | 5 |
| 144 | 5 |
| 148 | 5 |
| 152 | 5 |
| 156 | 5 |
| 160 | 5 |
| 164 | 5 |
| 168 | 5 |
| 172 | 5 |
| 176 | 5 |
| 180 | 5 |
| 184 | 5 |
| 188 | 5 |
| 192 | 5 |
| 196 | 5 |
| 198.668 | 5 |
| 198.668 | 5 |
| 200 | 5 |
| 200 | 5 |

***Supplementary Figure S1.*** *PEEP (cmH2O) as a function of leakage flow (lpm, 0–120) for three blower duty-cycle settings (DC% = 45%, 55%, 65%), simulated with Qpatient = 0.*

**Conclusion**
These results indicate that a duty-cycle setting of 55% is sufficient to maintain the desired PEEP level even in the presence of a physiological leakage flow up to 120 lpm, supporting the adoption of this setting as a robust operating condition for the device.
